# 3-T T2 mapping magnetic resonance imaging for biochemical assessment of normal and damaged glenoid cartilage: a prospective arthroscopy-controlled study

**DOI:** 10.1038/s41598-020-71311-4

**Published:** 2020-09-01

**Authors:** Felix Wuennemann, Laurent Kintzelé, Alexander Braun, Felix Zeifang, Michael W. Maier, Iris Burkholder, Marc-André Weber, Hans-Ulrich Kauczor, Christoph Rehnitz

**Affiliations:** 1 Diagnostic and Interventional Radiology, University Hospital Heidelberg, Im Neuenheimer Feld 110, 69120 Heidelberg, Germany; 2 Center for Orthopedics, Trauma Surgery and Spinal Cord Injury, University Hospital Heidelberg, Schlierbacher Landstraße 200A, 69118 Heidelberg, Germany; 3 Department of Nursing and Health, University of Applied Sciences of the Saarland, Saarbruecken, Germany; 4 Institute of Diagnostic and Interventional Radiology, Pediatric Radiology and Neuroradiology, University Medical Center Rostock, Ernst-Heydemann-Straße 6, 18057 Rostock, Germany; 5 Swabian Joint Center Stuttgart, ATOS Clinic Stuttgart, Hohenheimer Straße 91, 70184 Stuttgart, Germany

**Keywords:** Musculoskeletal abnormalities, Osteoarthritis

## Abstract

This study evaluated the ability of T2 mapping to assess the glenoid cartilage using arthroscopy as the gold standard. Eighteen consecutive patients (mean age: 52.4 ± 14.72 years, including 12 men) with shoulder pain underwent T2 mapping at 3-T with subsequent shoulder arthroscopy. With correlation to cartilage-sensitive morphologic sequences regions-of-interest were placed in the corresponding T2 maps both in normal-appearing cartilage and focal cartilage lesions using a quadrant-wise approach. Inter-reader and intra-reader correlation coefficients (ICCs) between two independent radiologists as well as cut-off values with their sensitivities/specificities for the detection of cartilage damage were calculated. The mean T2 value for healthy cartilage was 23.0 ± 3 ms with significantly higher values in the superior quadrants compared to the inferior quadrants (p < 0.0001). In 5 patients with focal cartilage damage significantly higher T2 values of 44.7 ± 3.7 ms (P < 0.01) were observed. The maximum T2 value in normal cartilage (27.3 ms) was lower than the minimum value in damaged cartilage (40.8 ms) resulting in perfect sensitivities/specificities of 100% (95% confidence-interval 47.8–100.0) for all cut-off values between 27.3–40.8 ms. ICCs ranged between 0.63 and 0.99. In conclusion, T2 mapping can evaluate biochemical cartilage integrity and discriminates arthroscopy-proven healthy and damaged glenoid cartilage with high diagnostic performance.

## Introduction

Osteoarthritis (OA) is a degenerative condition affecting the articulating facet joints. It is the most commonly encountered orthopaedic disorder and a leading cause of morbidity in elderly patients^1–3^. Although OA predominantly affects the weight-bearing joints, glenoid OA is a well-known cause of shoulder disability with an increasing incidence and prevalence and ultimately leads to endoprosthetic joint replacement^4, 5^. Cartilage defects are an important risk factor for development of OA^6, 7^. Therefore, timely diagnosis of early and potentially reversible disruption of the chondral architecture is desirable to delay the onset and progression of OA.

Magnetic resonance imaging (MRI) is the non-invasive gold standard for morphological evaluation of the articular cartilage^8^. However, early degenerative changes in the cartilage, such as loss of glycosaminoglycans and changes in water content, affect the ultrastructural integrity of the cartilage but are not readily detectable by conventional MRI ^9^. Development of functional MRI sequences like dGEMRIC (delayed gadolinium-enhanced MRI of the cartilage), T2* mapping, and T2 mapping has enabled assessment of the biochemical composition of the articular cartilage in various joints^7, 10–13^. T2 mapping is able to detect changes in the chondral collagen matrix and in the overall water content of the cartilage^9^. T2 mapping has been established and validated for detection and quantification of these early degenerative changes in various joints, including the knee, metacarpophalangeal joints, wrist, and ankle^7, 14–16^. Higher T2 values indicate damage to the three-dimensional collagen network and an increase in the water content^15–17^. However, T2 mapping has only rarely been used in the glenoid joint, and the technique has not been systematically validated using arthroscopy as the gold standard for normal or damaged cartilage in vivo. The few existing studies have focused on the feasibility of the technique in healthy or asymptomatic volunteers^18, 19^ without using a gold standard. One study examined the feasibility of glenoid T2 mapping in the context of primary and secondary OA; however, it included patients with more severe OA detected on conventional radiographs but not those with early degenerative changes. Therefore, patients in that study would have had severe cartilage damage because low-grade cartilage defects do not result in loss of chondral height and are not be visible on plain radiographs^20–22^. Furthermore, glenoid cartilage defects were not confirmed arthroscopically; therefore, disruption of the integrity of the cartilage was not confirmed using a gold standard method^14^.

The aim of this study was to assess the ability of T2 mapping to evaluate normal glenoid cartilage and detect focal cartilage defects in patients with shoulder pain but not high-grade OA using shoulder arthroscopy as the gold standard for comparative purposes.

## Results

### Demographic data

Twelve (66.7%) of the 18 patients enrolled were men and 6 (33.3%) were women. The mean patient age was 52.4 ± 14.72 (range, 22.0–67.0) years. The patients with normal-appearing cartilage were younger than those with cartilage lesions [48.6 ± 15.53 (range, 22–64) years vs 62.2 ± 5.54 (range, 53–67) years; P = 0.0245]. Table 1 summarises the demographic characteristics of the patients according to whether or not they had glenoid cartilage lesions.

**Table 1:** Demographic characteristics of patients with and without a glenoid cartilage lesion.

		Glenoid cartilage lesion		
		No (n = 13)	Yes (n = 5)	P-value (Wilcoxon test)
**Sex**	Male	8 (61.5%)	4 (80.0%)	
	Female	5 (38.5%)	1 (20.0%)	
**Age, years**	n	13	5	0.0245
	Mean	48.6	62.2	
	SD	15.53	5.54	
	Median	57.0	63.0	
	Min	22.0	53.0	
	Max	64.0	67.0	

*Max* maximum, *Min* minimum, *SD* standard deviation.

### Arthroscopic evaluation of the cartilage

Shoulder arthroscopy was performed in all patients. The median interval between MRI examination and surgery was 5 (range, 0–6) days. Glenoid cartilage damage was diagnosed in 5 patients (27.8%). According to the Outerbridge classification, one cartilage lesion was classified as grade 1 (20%), one as grade 2 (20%), one as grade 2–3 (20%), and two as grade 3 (40%). Two lesions were in the anteroinferior quadrant, one was in the anterosuperior quadrant, one extended from the anterosuperior quadrant to the anteroinferior quadrant, and one was predominantly in the anteroinferior quadrant but involved all quadrants.

### T2 mapping of normal, normal-appearing, and damaged glenoid cartilage

**Figure 1:**
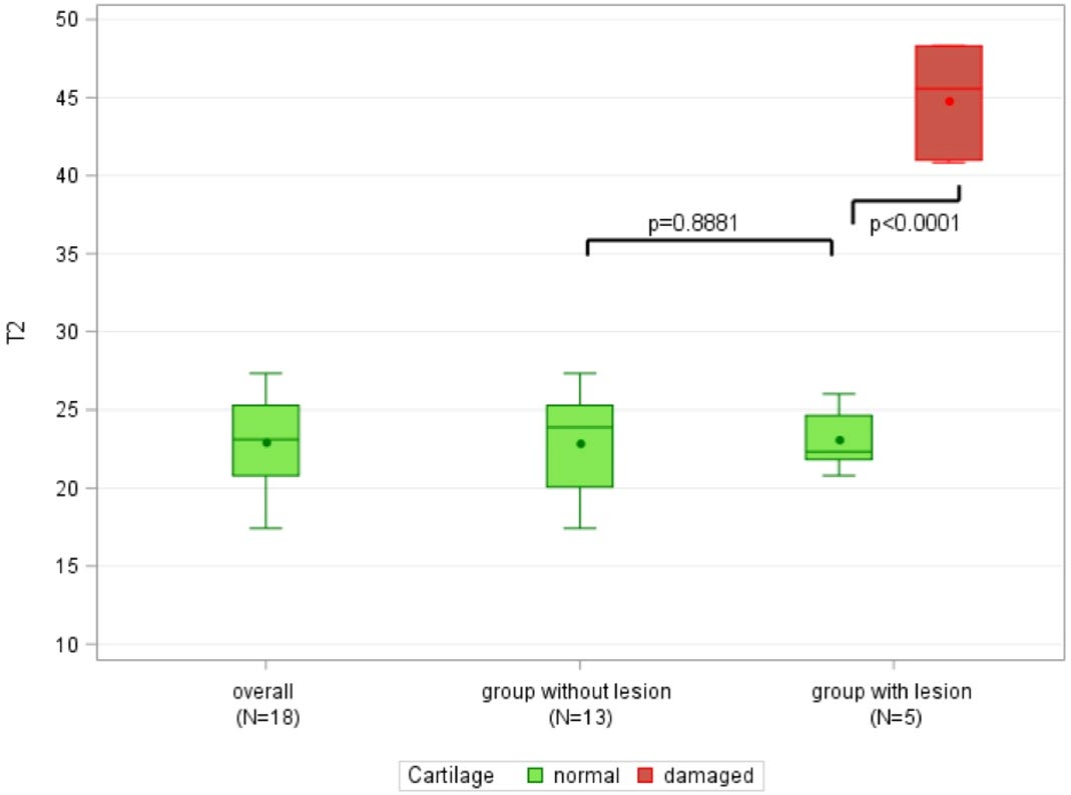
Boxplots of overall T2 mapping parameters in healthy, normal appearing, and damaged cartilage. Note the complete separation of the T2 mapping parameters between the damaged cartilage and the patients with healthy or normal-appearing cartilage.

Table 2 shows the mean T2 mapping parameters for healthy cartilage, for normal cartilage in the patient group without cartilage lesions, and for normal-appearing cartilage in patients with arthroscopically proven cartilage lesions, as well as the T2 mapping parameters of cartilage defects. Table 2 also depicts the T2 mapping parameters in the overall healthy cartilage as well as the subgroups of patients with normal cartilage in patients with arthroscopically proven healthy cartilage and normal-appearing cartilage in patients with confirmed cartilage lesions in the anterosuperior, anteroinferior, posterosuperior, and posteroinferior glenoid cartilage. The mean T2 mapping parameters for normal, healthy, and normal-appearing glenoid cartilage in the anterosuperior and posterosuperior quadrants were significantly higher than those in the inferior quadrants (P < 0.001). In contrast, there was no significant difference in the T2 mapping values between the anterosuperior and anteroposterior quadrants or between the anteroinferior and posteroinferior quadrants (P = 1.0). The mean overall T2 mapping value for normal cartilage was 23.0 ± 3 ms. There was no significant difference in the T2 mapping values between normal cartilage and normal-appearing cartilage in the patients with confirmed cartilage damage (22.3 ± 3.3 ms vs 23.1 ± 2.1 ms; P = 0.8881; Fig. 1).

**Table 2:** T2 mapping values for normal and damaged glenoid cartilage.

		Overall in healthy cartilage	Normal cartilage in population without lesions	Normal cartilage in population with lesions		Damaged cartilage
**Overall**	N	18	13	5		5
	Mean	23.0	22.9	23.1		44.8
	SD	3.0	3.3	2.1		3.7
	Median	23.1	23.9	22.3		45.6
	Min	17.4	17.4	20.8		40.8
	Max	27.3	27.3	26.0		48.3
**Anterior superior**	n	18	13	5		
	Mean	24.7	24.8	24.5		
	SD	3.0	3.4	1.2		
	Median	25.4	25.7	24.5		
	Min	19.1	19.1	20.8		
	Max	29.2	29.2	26.0		
**Anterior inferior**	n	18	13	5		
	Mean	21.6	21.6	21.4		
	SD	2.8	3.3	1.2		
	Median	22.1	22.9	21.8		
	Min	15.4	15.4	20.1		
	Max	25.5	25.5	22.5		
**Posterior superior**	N	18	13	5		
	Mean	24.4	24.2	25.0		
	SD	3.7	3.9	3.3		
	Median	24.4	23.9	24.9		
	Min	16.9	16.9	21.3		
	Max	28.9	28.9	28.7		
**Posterior inferior**	n	18	13	5		
	Mean	21.2	21.0	21.6		
	SD	3.4	3.4	3.6		
	Median	20.6	20.7	20.6		
	Min	15.4	15.4	18.2		
	Max	26.9	26.5	26.9		

*Max* maximum, Min minimum, *SD* standard deviation.

Five lesions were detected on MRI. These lesions corresponded to the arthroscopic lesions. No additional lesions were found. Table 3 shows the lesion grading for each lesion, the mean T2 mapping value per lesion, and the average T2 mapping value for normal-appearing cartilage in the respective quadrants. The mean T2 mapping value for damaged cartilage was 44.8 ± 3.7 ms, resulting in a significant difference between normal and damaged cartilage (P < 0.0001). There was a complete separation of the T2 mapping values between normal or normal-appearing cartilage and the T2 mapping values for damaged cartilage, meaning that the highest T2 mapping values for normal or normal-appearing cartilage were lower than the lowest T2 mapping values in the group with confirmed cartilage damage (27.3 ms in the patient group without cartilage damage, 26.0 ms in the normal-appearing cartilage in patients with confirmed cartilage lesions, and 40.8 ms in those with cartilage damage). These findings persisted when comparing the lowest T2 mapping value for damaged cartilage with the highest T2 mapping values in the individual quadrants (26.0 ms in the anterosuperior quadrant, 22.5 ms in the anteroinferior quadrant, 28.9 ms in the posterosuperior quadrant, and 26.5 ms in the posteroinferior quadrant).

**Table 3:** Characteristics of individual focal cartilage lesions.

Lesion	Location	Outerbridge grade	Mean T2 mapping parameter of lesion	Mean T2 mapping of normal cartilage in respective quadrant
1	Anteroinferior	1	45.6	21.6
2	Anteroinferior	3	48.3	21.6
3	Anterosuperior	3	40.8	24.7
4	Predominantly anterosuperior	2	41.0	24.7
5	Predominantly anteroinferior	2–3	48.3	21.6

### T2 mapping measurements: inter-reader and intra-reader agreement

Intra-class correlation coefficient (ICC) analysis revealed near-perfect inter-reader agreement for both normal and damaged cartilage (Table 4). The inter-reader agreement for normal cartilage was 0.99 (95% confidence interval [CI] 0.97–0.97) and for damaged cartilage 0.92 (95% CI 0.49–0.99). The intra-reader agreement for normal cartilage, calculated in all quadrants, was almost perfect, with an ICC ranging between 0.94 and 0.97 for reader 1 and between 0.87 and 0.96 for reader 2. The intra-reader agreement in cartilage lesions was moderate with ICCs of 0.63 (95% CI 0.29–0.83) for reader 1 and 0.69 (95% CI 0.13–0.92) for reader 2 respectively.

**Table 4:** Inter-reader and intra-reader agreement.

ICC	Estimate	95% confidence interval
Inter-reader agreement—normal cartilage	0.99	[0.97–0.99]
Inter-reader agreement—pathological cartilage	0.92	[0.49–0.99]
Intra-reader agreement—normal cartilage Reader 1—AS Intra-reader agreement—normal cartilage Reader 1—AI Intra-reader agreement—normal cartilage Reader 1—PS Intra-reader agreement—normal cartilage Reader 1—PI	0.94 0.94 0.94 0.97	[0.91–0.96] [0.91–0.96] [0.91–0.96] [0.95–0.98]
Intra-reader agreement—pathological cartilage Reader 1	0.63	[0.29–0.83]
Intra-reader agreement—normal cartilage Reader 2—AS Intra-reader agreement—normal cartilage Reader 2—AI Intra-reader agreement—normal cartilage Reader 2—PS Intra-reader agreement—normal cartilage Reader 2—PI	0.87 0.92 0.93 0.96	[0.76–0.93] [0.84–0.96] [0:87–0.96] [0.92–0.98]
Intra-reader agreement—pathological cartilage Reader 2	0.69	[0.13–0.92]

AI anterior inferior, *AS* anterior superior, *ICC* intraclass correlation, *PI* posterior inferior, *PS* posterior superior.

## Discussion

Following cardiovascular disease and cancer, OA is among the leading causes of morbidity in an aging society. Chondral degeneration is one of the most important risk factors for articular degeneration. Therefore, early diagnosis of cartilage defects is desirable to delay the onset and progression of the disease, which often requires joint replacement^4, 5^. The literature on functional imaging of the glenoid cartilage is limited and, to the best of our knowledge, there has only been one study examining T2 mapping of the glenoid joint in advanced OA^14^.

In this study, we evaluated the ability of T2 mapping to detect glenoid cartilage defects using shoulder arthroscopy as the control. The T2 mapping values for normal-appearing cartilage in the superior portions of the glenoid cartilage were significantly than those in the inferior quadrants (P < 0.001). These findings are in line with observations by Lee et al. and Bittersohl et al., who also reported lower T2 mapping values in the inferior portions of glenoid cartilage that were confirmed arthroscopically to be normal^13, 14^. In our patient population, the mean T2 mapping value for glenoid cartilage was 23. ± 3.0 ms. In contrast, Kang and Choi reported a mean T2 mapping value of 49.0 ± 9.9 ms in their study of healthy volunteers^19^. A further study performed by Lee et al. and controlled by radiography and MRI found a T2 mapping value of 36 ms in patients without OA^14^. However, in contrast with our study, arthroscopic evaluation was not included in either of the above-mentioned studies. Therefore, the gold standard for confirmation of healthy cartilage was missing. Many factors have been reported to affect T2 mapping values. Therefore, differences in the T2 mapping value might be explained by differences in the MRI systems used, coil setup, patient age, spatial variation, the T2 magic angle effect, and location of the cartilage assessed ^23, 24^. Furthermore, Mars et al. reported differences in T2 values depending on the type of T2 map and calculation method^25^. To conquer these problems, future studies may not only compare absolute T2 mapping values of normal and damaged cartilage but also focus on relative measurements, such as the difference between normal and damaged cartilage or relative T2 mapping indices (e.g. T2_damaged cartilage_ divided by T2_healthy cartilage_) in order to increase the comparability of these studies.

There was no significant difference in the T2 mapping values between the normal cartilage in patients with arthroscopically confirmed normal cartilage and morphologically normal-appearing cartilage in patients with cartilage lesions confirmed by MRI (22.9 ± 3.3 ms vs 23.1 ± 2.1 ms; P = 0.881). This finding suggests that early cartilage damage might be a localised process. The progression of focal articular cartilage damage to degenerative OA is not well understood. Guettler et al. reported a size-related increase in rim stress and redistribution of load in osteochondral lesions of the femoral condyle that were greater than 10 mm in diameter ^26^. However, there are no similar studies investigating the progression of focal cartilage defects to degenerative OA of the glenoid joint.

In our study, patients with glenoid cartilage defects had a significantly higher mean T2 mapping value than those without cartilage defects (44.8 ± 3.7 ms vs. 23.0 ± 3.0 ms, respectively). This finding may reflect ultrastructural disruption of the chondral collagen network and an increased water content in cartilage with early degenerative changes^9^. The increase in T2 mapping values in areas of damaged cartilage is in accordance with the findings of Lee et al., who reported that T2 mapping values were higher in patients with radiographically diagnosed primary glenoid OA than in those without OA ^14^. Furthermore, our findings are in line with those of T2 mapping studies of OA in other joints that reported increased T2 mapping values in damaged cartilage^17, 27, 28^. In our study population, the highest overall T2 mapping value in normal cartilage in healthy patients (27.3 ms) and in normal-appearing cartilage in the patients with proven cartilage damage (26.0 ms) were lower than the lowest overall T2 value in the patients with cartilage damage (40.8 ms), indicating complete separation of the data in these study groups. Theoretically, all the cut-off T2 mapping values would result in sensitivity, specificity, negative predictive, and positive predictive values of 100% (95% CI 47.8–100). Although significant differences in T2 mapping values between normal and damaged cartilage have been described before^7, 14, 17, 27–29^, complete separation of T2 values has never been described. The T2 mapping values in cartilage are influenced by a variety of factors, including the orientation of the collagen network relative to the magnetic field, the so-called “magic angle” effect resulting in increased signal intensity, compositional changes related to the mechanical load, and the water content^13, 14, 17, 23^. T2 mapping sequences are susceptible to the magic angle effect that results in an increase of the T2 relaxation time in cartilage fibers oriented 55° to the magnetic field^30–32^. In fact, Mosher et al. reported a T2 relaxation time increase of up to 29% in the superficial layer of the femoral cartilage at 55° orientation^30^. Although literature of the T2 mapping magic angle effect on glenoid cartilage is missing, an increase of the T2 relaxation time in the given angle can be expected. However, the complete separation of data persisted when the lowest T2 mapping value of the damaged cartilage was compared with the highest T2 mapping value in any given quadrant. Therefore, we assume that the complete separation of the data was not caused by a blurring of data because the T2 mapping parameters were averaged for all four quadrants and the regions of interest were drawn in a way that covers the whole curvature of the glenoid cartilage in a given quadrant which should decrease the effect of the magic angle on the averaged T2 relaxation time. Our present findings might be attributed to careful placement of regions of interest (ROIs) as well as a small study population with only five cartilage defects.

T2 mapping values for normal and damaged cartilage showed near-perfect inter-reader agreement, with respective ICCs of 0.99 (95% CI 0.97–99) and 0.92 (95% CI 0.49–0.99), further underlining the robustness of this technique. This good to excellent inter-reader agreement is in line with previously published studies of T2 mapping of the glenoid joint in healthy volunteers^19, 33^. Quadrant-wise calculation of intra-reader agreement showed a good to near-perfect ICC for normal and normal-appearing cartilage (0.94–0.97 for reader 1 and 0.87–0.96 for reader 2 respectively). However, the intra-reader agreement for damaged cartilage was moderate, with an ICC of 0.63 (95% CI 0.29–0.83) for reader 1 and 0.69 (95% CI 0.13–0.92) for reader 2. Given the broad 95% confidence interval, we believe that the moderate intra-reader agreement is caused mainly by the low number of cartilage defects (only 5) but increased further by the various biochemical processes that occur during chondral damage; these are not homogeneously distributed in the cartilage defect, resulting in some expected variation in T2 mapping values. Surprisingly, the intra-reader agreement for cartilage lesions was lower than the respective inter-reader agreement. This disparity might be explained by the aforementioned concurring biochemical processes in damaged cartilage and the small sample size. However, this finding might also be attributed to the methodological shortcomings of a manual region-of-interest placement which is prone to human error highlighting the need for semi-automated or automated cartilage evaluation methods that need to be addressed in future studies.

Our study has some limitations. First, the sample size was small which may have contributed to the complete separation of T2 values between the damaged and normal or normal-appearing cartilage, leading to statistically perfect discrimination using T2 mapping. Although increased T2 mapping values can be expected in damaged cartilage, further studies with larger sample sizes are required to confirm these findings and to evaluate a possible overlap of values. Furthermore, the subgroup with damaged cartilage was too small to differentiate between patients with grade 1, 2, and 3 defects in a statistical valid manner. However, we have provided the T2 values for each cartilage defect in a descriptive one-on-one fashion. Given the above-mentioned variations in T2 mapping values, our findings might only be applicable to our patient cohort. Moreover, there was a significant difference in age range between our group with healthy cartilage and that with damaged cartilage. However, there was no significant difference in T2 mapping values between the normal and normal-appearing cartilage. This limitation needs to be addressed in future research. Further studies that include a larger sample size and a wider age range are needed to confirm our findings. Furthermore, we evaluated cartilage defects using morphological correlates on conventional MRI. We acknowledge that our assessment method using a manually drawn ROI on a two-dimensional sequence is not perfect. The sequence used in this study is a product sequence of the manufacturer. It provided robust results in a comparably short imaging and post-processing time. However, as a task for future studies, a technically more advanced approach using a high resolution three-dimensional T2-mapping sequence possibly with automatic assessment of the entire cartilage volume and a voxel-by-voxel approach with normalised geometry would be desirable. Therefore, future studies might benefit from use of three-dimensional T2 mapping sequences allowing for a voxel-by-voxel evaluation of the T2 mapping parameters. As a next step, a detailed investigation of normal-appearing cartilage with deviating T2 mapping values is needed to determine the ability of T2 mapping to detect very early cartilage defects and to assess the effect of early conservative measures, such as physiotherapy.

The ability to assess early changes in cartilage composition may have implications for patient care and aid in therapeutic decision-making in several shoulder pathologies. This might be of considerably interest when counselling patients prone to early glenoid damage, e.g., overhead athletes^34^. Furthermore, rotator cuff tears lead to premature cartilage damage^35, 36^. However, the status of cartilage is not routinely assessed at present, and it is unclear which patients should undergo surgical repair in order to prevent cartilage damage/osteoarthritis. For example, it has been reported that microinstability may lead to many shoulder injuries, including cartilage defects, in overhead athletes^37, 38^. In these patient groups, T2 mapping may be used to aid therapy decision-making and to identify patient subgroups that would benefit from surgical therapy. Modern cartilage therapy can be used to repair cartilage defects in the shoulder. The ability of T2 mapping to evaluate repair of cartilage has been established for other joints^7, 39^. Finally, T2 mapping may be used as a baseline for any biochemical therapy that may be developed in the future.

In conclusion, T2 mapping can detect and quantify arthroscopically confirmed healthy glenoid cartilage and differentiates cartilage lesions with a high diagnostic performance value. T2 mapping may be used to evaluate the biochemical integrity of glenoid cartilage and may aid in therapeutic decision-making in various shoulder pathologies.

## Methods

### Subjects

The study was approved by the Institutional Review Board of the University of Heidelberg (S-081/2010) and performed in accordance with the Declaration of Helsinki. Written informed consent was obtained from all patients after the nature of the examination had been explained. Patients with shoulder pain scheduled for shoulder arthroscopy who were referred to our department for MRI during a 3-month period were considered for enrolment in the study. Patients with known advanced OA (Kellgren-Lawrence score > 1), those with a shoulder endoprosthesis, and those younger than 18 years of age were excluded. Nineteen consecutive patients met the inclusion criteria and were enrolled in the study. A further patient who underwent arthroscopy and in whom a routine MRI protocol was inadvertently performed without T2 mapping sequences was also excluded, leaving 18 patients for inclusion in the study.

### MRI protocol and T2 mapping

All patients were examined using a 70-cm open bore 3-T whole-body MR scanner (Magnetom Verio, Siemens Healthineers, Erlangen Germany) with an 18-channel total imaging matrix (Tim [102 × 18] configuration) and a 4-channel transmit-receive flex coil (Siemens Healthineers). The patients were positioned head-first and supine with the shoulder joint externally rotated. The shoulder was positioned as close as possible to the isocentre of the magnet. Our radiographers were advised to stabilise the shoulder joint in order to reduce or prevent movement artefacts. The morphology of the shoulder was assessed using our in-house standard shoulder MRI protocol, which included cartilage-sensitive proton-density weighted fat saturated sequences in all three planes for morphologic cartilage assessment. Coronal sequences were acquired with an oblique coronal orientation perpendicular to the glenoid fossa. Consequently, sagittal sequences were acquired with an oblique sagittal orientation parallel to the glenoid fossa. In addition, coronal and axial T2 mapping was performed using the vendor supplied multiecho spin-echo T2 weighted mapping sequence (syngo MapIT, Siemens Healthineers, Erlangen, Germany), which provides the protocols and automatically calculates parametric color-coded maps. T2 relaxation times for further analysis were derived from the T2 parameters by pixel-wise monoexponentional least-squares-fit analysis (syngo MapIT, Siemens Healthineers, Erlangen, Germany). Table 5 gives detailed information about the standard in-house shoulder protocol as well as the axial and coronal T2 mapping sequences used in this study.

**Table 5:** In-house shoulder MRI protocol and T2 mapping study sequences.

No.	Sequence	Orientation	Repetition time (TR; ms)	Echotime (TE; ms)	Voxelsize (mm)	Acquisition matrix	Flip angle	Echo train length	No. of slices	TA (min)
1	PD fs TSE	Axial	3660	24	0.5 × 0.5 × 3	384 × 346	176	7	27	04:32
2	PD fs TSE	Oblique coronal	2490	24	0.5 × 0.5 × 3	384 × 307	160	7	19	03:37
3	PD fs TSE	Oblique sagittal	3950	23	0.6 × 0.6 × 3	320 × 256	140	7	29	04:49
4	PD TSE	Oblique coronal	1670	23	0.5 × 0.5 × 3	384 × 307	160	5	19	03:24
5	T1 SE	Oblique coronal	787	10	0.5 × 0.5 × 3	384 × 346	90	1	19	04:51
6	T2 TSE	Oblique sagittal	5640	88	0.5 × 0.5 × 3	384 × 307	150	15	29	02:33
7	T2 MapIt	Axial	2140	13.8, 27.6, 41.4, 55.2, 69	0.6 × 0.6 × 3	256 × 256	180	1	17	04:00
8	T2 MapIt	Oblique coronal	2140	13.8, 27.6, 41.4, 55.2, 69		320 × 320	180	1	16	06:50

### Image analysis and definition of cartilage damage

The morphological images were analysed using a picture archiving and communication system (Centricity PACS, v. 4.0, GE Healthcare IT Solutions, Barrington, IL, USA). All studies that included color-coded parametric T2 maps were evaluated separately by two musculoskeletal radiologists with 13 years (CR) and 4 years (FW) of experience in musculoskeletal MRI. Both readers have gained experience in evaluation of cartilage in previous studies including quantitative biochemical cartilage imaging techniques. Moreover, both readers have undergone previous training sessions involving cases that were not included in this study. Both radiologists determined the slice selection, magnification, and windowing parameters. Ambient light was kept to a minimum during the reading session.

**Figure 2:**
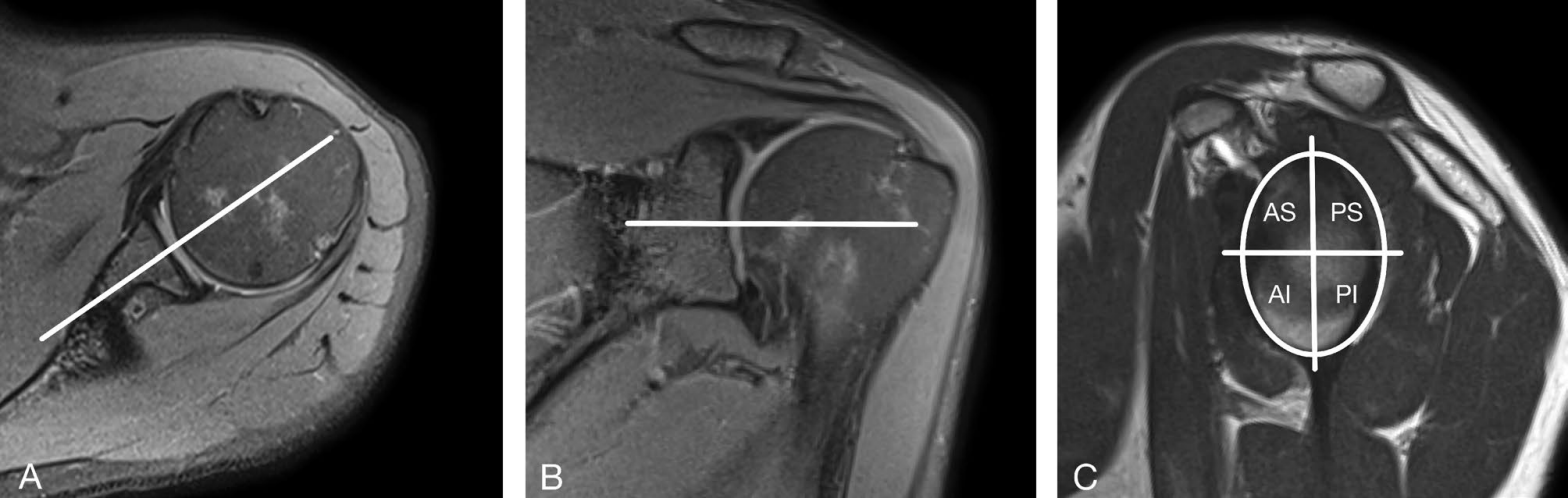
**(A)** Axial proton-density fat saturated magnetic resonance image used to divide the glenoid cartilage in anterior and posterior segments. **(B) **Coronal proton-density fat-saturated magnetic image used to divide the glenoid cartilage in superior and inferior segments. **(C)** Sagittal T2-weighted magnetic resonance image with a depiction of the four-quadrant approach. By halving the glenoid cartilage on axial and coronal images (white lines in **A** and **B**) the glenoid cartilage was divided into and anterosuperior (AS), posterosuperior (PS), anteroinferior (AI) and posteroinferior (PI) quadrant.

The glenoid cartilage was subdivided into anterosuperior, anteroinferior, posterosuperior, and posteroinferior quadrant quadrants for systematic analysis of the images. The superoinferior borderline was placed by halving the glenoid on midcoronal images and the anteroposterior borderline by halving the glenoid on axial images^40^ (Fig. 2). Cartilage was classified as normal or damaged using the modified Outerbridge method^41^. Using morphological proton density-weighted fat-saturated sequences, damaged cartilage was defined as a focal superficial or deep defect, thinning of the entire chondral layer, or focal swelling with oedema and irregularity corresponding to a modified Outerbridge grade of 1–3. Full-thickness cartilage lesions corresponding to modified Outerbridge grade 4 were not analysed because of a lack of measurable cartilage^41^. Cartilage without the aforementioned diagnostic criteria was considered to be normal or normal-appearing. Each cartilage defect was localised by quadrant^40^. A cartilage defect extending to more than one quadrant was counted as one cartilage lesion and assigned to the quadrant containing the largest cartilage defect.

To obtain the most accurate measurements, ROIs were placed in the first-echo images acquired using a multi-echo-spin-echo T2-weighted sequence, which enabled ready delineation of opposing cartilage layers as well as the subchondral endplate. A meticulous visual comparison using the cartilage-sensitive fat-suppressed proton density-weighted sequences was performed to facilitate optimal ROI placement and to ensure that the entire cartilage defect was covered. The ROIs were then copied into the color-coded parametric T2 maps. If necessary, the copied ROIs were adjusted to ensure that the final ROI did not contain any artefacts, the subchondral bone plate, or joint fluid. In patients with cartilage defects, the ROIs in normal-appearing cartilage were placed in the opposing quadrant of the same slice, covering the largest possible area without contact with the area of cartilage damage.

**Figure 3:**
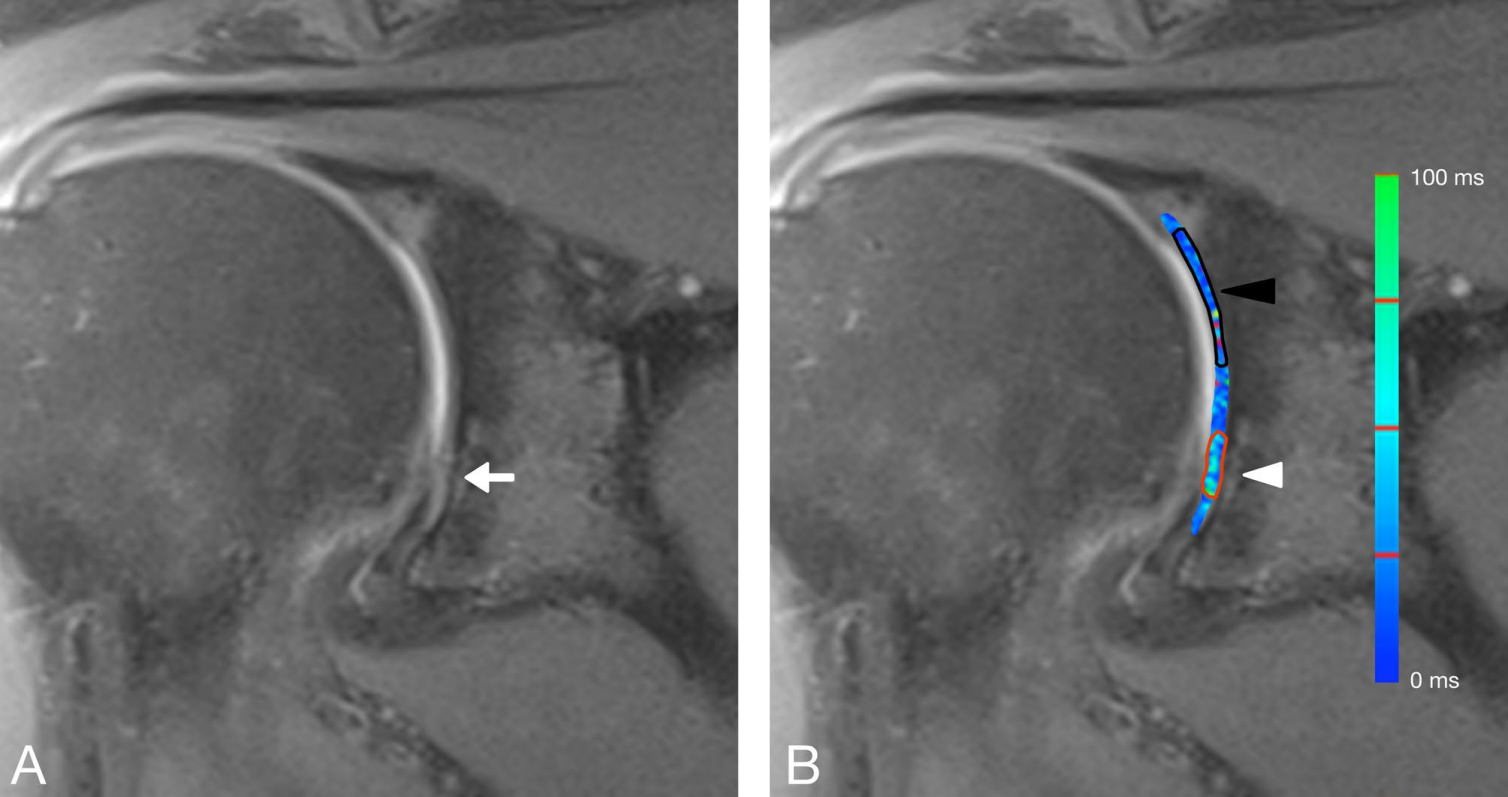
(**A**) shows a coronal proton-density weighted fat saturated magnetic resonance image of a 53-year-old male patient with a focal anteroinferior cartilage lesion (white arrow). (**B**) shows a merged image of the coronal proton density fat-saturated section and the corresponding color-coded T2 map. The average T2 mapping value in the area of the focal cartilage defect (white arrowhead, red ROI) was 45.67 ms (Reader 1, Read 1) whereas that for the normal-appearing cartilage in the anterosuperior quadrant (black arrowhead, black ROI) was 22.7 ms (Reader 1, Read 1). The average T2 mapping value of normal cartilage in the anteroinferior quadrant (ROI not shown) was 20.43 ms (Reader 1, Read 1).

**Figure 4:**
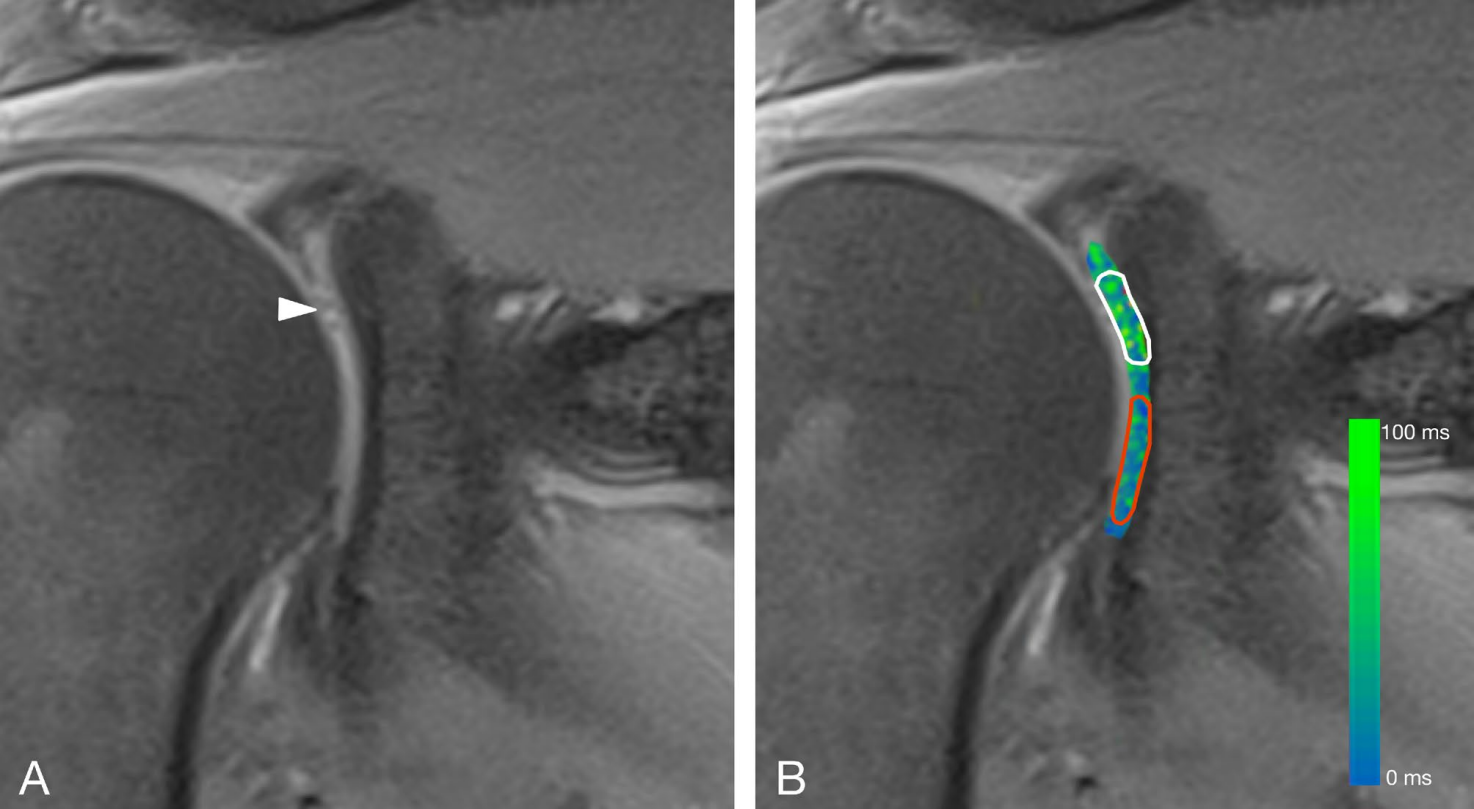
**(A)** Coronal proton-density fat saturated magnetic resonance image of a 55-year old female patient with a focal cartilage defect in the anterosuperior quadrant (white arrowhead). **(B)** Coronal proton-density fat saturated magnetic resonance image merged with the corresponding color-coded T2 map. The average T2 mapping value in the area of the focal cartilage defect was 40.8 ms (white ROI). The average T2 mapping value in the anteroinferior glenoid cartilage (red ROI) was 22.1 ms (Reader 1, Read 1). The average T2 mapping value of normal cartilage in the anterosuperior quadrant (ROI not shown) was 25.73 ms (Reader 1, Read 1).

The T2 relaxation times of normal cartilage were measured in each quadrant. As suggested in previous studies in healthy volunteers, ROIs in normal cartilage were placed in the coronal section with the best visibility of the chondral layer^19^. In quadrants without visible cartilage defects, the size of the ROI was set to be as large as possible, covering the entire glenoid cartilage without including visible artefacts, joint fluid, or the subchondral bone plate 19. In patients with glenoid cartilage defects, the ROI for measurement of normal cartilage in the affected quadrant was placed with the size as large as possible. A thorough visual comparison with the cartilage-sensitive proton density-weighted sequences was performed to ensure that the ROI did not contain any areas of damaged cartilage (Figs. 3 and 4).

To reduce the effect of artefacts or incorrectly placed ROIs, each ROI placement was repeated three times and the average T2 relaxation time was used for further analysis^7^.

To analyse the intra-reader correlation, reader 1 (RC) and reader 2 (FW) repeated all measurements with an interval between reading sessions of 7 days to minimise the repetition bias. Both readers gained experience in evaluation of cartilage, precise placement of ROIs, and identification and avoidance of the artefacts in previous studies.

### Statistical analysis

The patient demographics were analysed descriptively. Continuous variables were summarised as the mean, standard deviation, median, minimum, and maximum. Qualitative variables were analysed by calculating the frequency and percentage. The age of the patients with and without lesions was compared using the Wilcoxon two-sample test.

Three measurements of healthy and damaged cartilage were performed by the two readers for analysis of the T2 mapping parameters. Both readers repeated the measurements with a time interval of 7 days for calculation of intra-reader agreement. The normal-appearing cartilage was measured in the above-mentioned four quadrants. Therefore, nine measurements for each patient and each section were available for statistical analysis. Analysis of T2 imaging was descriptive using summary statistics and was interpreted in an exploratory manner. Summary statistics were calculated for healthy cartilage separately for the four sections using the mean of nine measurements per patient and overall using the mean of all 36 measurements per patient. Summary statistics were calculated using the mean of all nine measurements per patient for pathological cartilage. The Shapiro–Wilk test was used to assess whether the T2 mapping parameters were normally distributed or not. Measurements in normal and pathological cartilage were compared using the paired *t*-test. Groups with and without lesions were compared using the two-samples *t*-test. Furthermore, differences between the four sections were analysed using analysis of variance. Pairwise comparisons between sections were evaluated using the paired *t*-test with Bonferroni adjustment. The level of significance was set to 5%. All statistical analyses were performed using SAS for Windows version 9.4 (SAS Institute Inc., Cary, NC, USA) and R version 3.5.1 (https://www.cran.r-project.org).

The ICC was used to quantify the inter-reader and intra-reader agreement. The two readers were considered as a random sample of observers from a larger population of potential observers. In a study of inter-reader reliability by Shrout and Fleiss, a two-way random-effects model with subject and reader as random effects was applied for estimation of ICCs and 95% confidence intervals^42^. The mean of multiple reads per person and reader was used to calculate the ICCs. For intra-reader reliability, the ICC and 95% confidence interval were calculated for each reader using a two-way mixed-effect model.

### Arthroscopy

Shoulder arthroscopy was performed by either of two experienced orthopaedic surgeons (FZ or MM). The arthroscopies were performed under general intravenous anaesthesia with all patients placed in the beach-chair position. A posterior approach was used for diagnostic inspection of the glenoid joint. The glenoid cartilage was evaluated using a standardised questionnaire. Cartilage lesions were graded using the Outerbridge classification^41^. As proposed by Jaeger et al.^40^, the glenoid fossa was subdivided into four quadrants (anterosuperior, anteroinferior, posterosuperior, posteroinferior) to localise the cartilage defects. If a surgical intervention was necessary, a ventral approach was established.

## Data Availability

The datasets generated during and/or analysed during the current study are available from the corresponding author on reasonable request.
